# Rab6a enables BICD2/dynein-mediated trafficking of human papillomavirus from the *trans-*Golgi network during virus entry

**DOI:** 10.1128/mbio.02811-24

**Published:** 2024-10-21

**Authors:** Jeongjoon Choi, Kaitlyn Speckhart, Billy Tsai, Daniel DiMaio

**Affiliations:** 1 Department of Genetics, Yale School of Medicine, New Haven, Connecticut, USA; 2 Department of Cell and Developmental Biology, University of Michigan Medical School, Ann Arbor, Michigan, USA; 3 Cellular and Molecular Biology Program, University of Michigan Medical School, Ann Arbor, Michigan, USA; 4 Department of Therapeutic Radiology, Yale School of Medicine, New Haven, Connecticut, USA; 5 Department of Molecular Biophysics & Biochemistry, Yale University, New Haven, Connecticut, USA; 6 Yale Cancer Center, New Haven, Connecticut, USA; The University of North Carolina at Chapel Hill, Chapel Hill, North Carolina, USA

**Keywords:** Rab6a, BICD2, HPV, dynein, virus trafficking, *trans*-Golgi network, L2 protein, retrograde transport, Rab33b

## Abstract

Rab GTPases control intracellular vesicular transport, including retrograde trafficking of human papillomavirus (HPV) during cell entry, guiding the virus from the endosome to the *trans-*Golgi network (TGN), the Golgi apparatus, and eventually the nucleus. Rab proteins have been identified that act prior to the arrival of HPV at the TGN, but Rab proteins operating in later stages of entry remain elusive. Here, we report that knockdown of Rab6a impairs HPV entry by preventing HPV exit from the TGN and impeding intra-Golgi transport of the incoming virus. Rab6a supports HPV trafficking by facilitating the association of HPV with dynein, a motor protein complex, and BICD2, a dynein adaptor, in the TGN. L2 can bind directly to GTP-Rab6a *in vitro*, and excess of either GTP-Rab6a or GDP-Rab6 inhibits HPV entry, suggesting that cycling between GDP-Rab6 and GTP-Rab6 is critical. Notably, Rab6a is crucial for HPV-BICD2 and HPV-dynein association in the TGN of infected cells but not in the endosome. Our findings reveal important features of the molecular basis of HPV infection, including the discovery that HPV uses different mechanisms to engage dynein at different times during entry, and identify potential targets for therapeutic approaches to inhibit HPV infection.

**IMPORTANCE:**

Human papillomaviruses (HPVs) are small, non-enveloped DNA viruses that cause approximately 5% of human cancer. Like most other DNA viruses, HPV traffics to the nucleus during virus entry to successfully infect cells. We show here that HPV utilizes a cellular enzyme, Rab6a, during virus entry to engage the dynein molecular motor for transport along microtubules. Rab6a is required for complex formation between the HPV L2 capsid protein, dynein, and the dynein adaptor BICD2 in the *trans*-Golgi network (TGN). This complex is required for transport of the incoming virus out of the TGN as it journeys to the nucleus. Our findings identify potential targets for therapeutic approaches.

## INTRODUCTION

Rab GTPases control intracellular vesicular transport of cellular proteins (1–3). Rab proteins also play critical roles in viral infections, including infection by human papillomaviruses (HPVs) (4–7). HPVs are non-enveloped, double-stranded DNA viruses that are responsible for 5% of human cancer, including essentially all cervical cancers (8). Several Rab proteins are involved in HPV entry (6), some of which have been assigned specific roles in the entry process. Rab9a and Rab7 act at relatively early steps in this process, prior to the arrival of HPV at the *trans-*Golgi network (TGN) (9–11). Here, we show that Rab6a acts in a relatively late phase of HPV trafficking during virus entry, by coupling HPV to dynein to allow HPV to exit from the TGN.

The HPV capsid is composed of 360 molecules of the major capsid protein, L1, and up to 72 molecules of the minor capsid protein, L2 (12). L1 is responsible for HPV binding to the cell surface, while L2 is critical for the trafficking of the viral genome to the nucleus where viral DNA (vDNA) replication and viral gene expression occur (13, 14). After internalization of HPV, viral components including vDNA reside within vesicular retrograde trafficking compartments throughout the entry process as HPV is transported from endosome to the TGN, through the Golgi apparatus and possibly the endoplasmic reticulum (ER) en route to the nucleus (15–19). After initially localizing in endosomes, endosome acidification and γ-secretase activity allow a C-terminal cell-penetrating peptide (CPP) on the L2 capsid protein to drive a segment of L2 across the endosomal membrane to protrude into the cytosol, so that L2 can interact with various cytoplasmic host factors to enable retrograde trafficking of the incoming virus particle (20, 21). L2 uses this mechanism to associate directly with multiple trafficking factors including retromer (22), sorting nexins 17 and 27 (23, 24), dynein (25), retriever (26), and COPI (27). L2 also associates directly or indirectly with Rab9a (11) and Rab7 (10, 11). Most of these factors are involved in relatively early stages of HPV entry, from endosome-to-TGN trafficking of the virus. How HPV is transported from the TGN to the nucleus is poorly understood.

Dynein, a multi-protein complex, plays a pivotal role in orchestrating the intracellular transport of membrane vesicles along microtubules. Dynein associates with L2 during entry, and HPV entry is impaired by chemical inhibition of dynein, mutations in L2 that impair its interaction with dynein, or knockdown of dynein component(s) (25, 28, 29). In addition, RanBP10, a dynein adaptor, facilitates the nuclear import of the HPV vDNA-L2 complex (29). However, other than this late nuclear import step in entry, it remains largely unclear how dynein acts to support other steps of HPV entry. We recently reported that another dynein adaptor, BICD2, promotes HPV trafficking from endosome to TGN and beyond (30). HPV L2 can bind directly to BICD2, and knockdown of BICD2 inhibits the HPV-dynein interaction and results in HPV accumulation in endosomes and the TGN.

The role of Rab6a in HPV infection is controversial. An early study involving overexpression of wild-type and mutant Rab6a did not reveal a requirement for Rab6a in HPV pseudovirus (PsV) infection (31), whereas our small interfering RNA (siRNA) screen revealed that infection was inhibited by knockdown of Rab6a and Rab6IP1, a Rab6a guanine nucleotide exchange factor (15). In this study, we show that Rab6a supports HPV type 16 (HPV16) PsV entry by binding to L2 and recruiting HPV to dynein. Unlike other Rab proteins, which associate with HPV in endosomes, Rab6a engages HPV later during entry in the TGN, suggesting that Rab6a acts at relatively late stages in the intracellular journey of HPV. Consistent with this view, HPV-BICD2 association and HPV-dynein association require Rab6a at 16 h post-infection (hpi) but not at earlier times, and Rab6a is critical for exit of HPV from the TGN during entry. An excess of either GTP- or GDP-bound forms of Rab6a impairs HPV infection, suggesting that cycling of Rab6a is critical for HPV entry.

## RESULTS

### Rab6a is required for HPV egress from *trans*-Golgi during HPV entry

**Fig 1:**
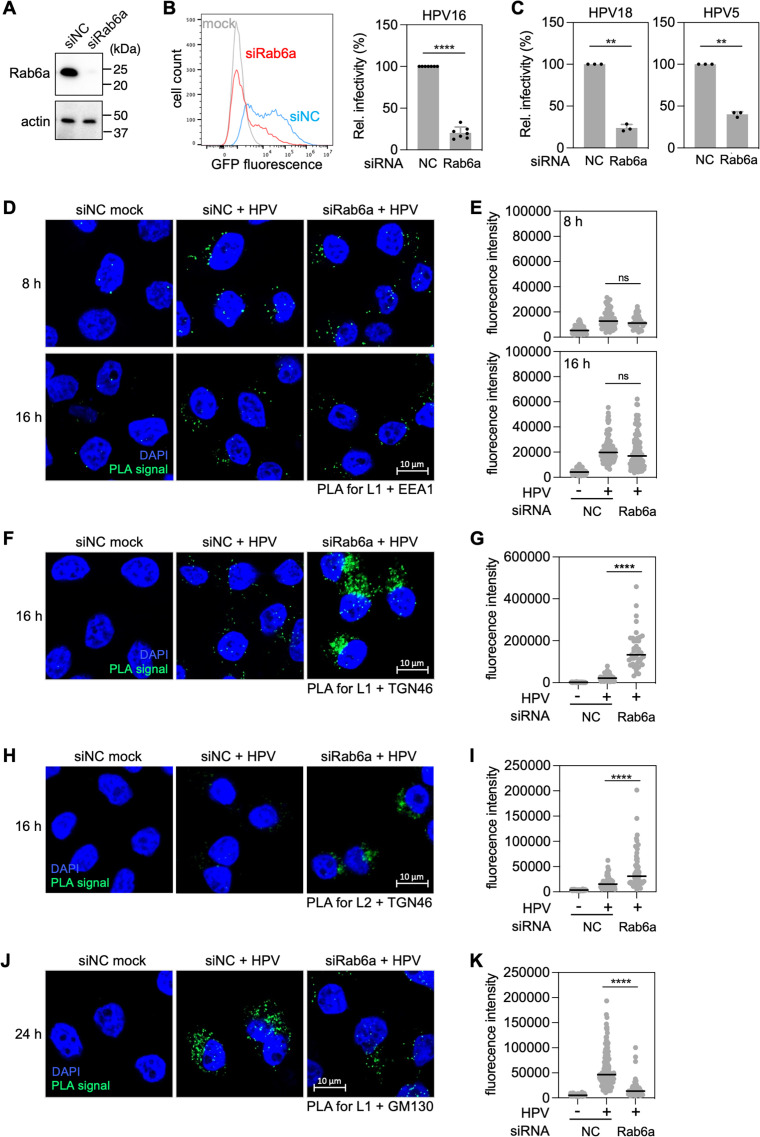
Rab6a knockdown inhibits HPV infection by impairing trafficking from *trans-*Golgi network to the Golgi stack. (**A**) HeLa cells were transfected with negative control (siNC) or Rab6a-targeting siRNA (siRab6a) and subjected to western blot analysis using an antibody recognizing Rab6a (top panel) or actin (as a loading control, bottom panel). (**B**) siRNA-treated cells as described in panel **A** were mock-infected or infected at the MOI of ~1 with HPV16 PsV L2-3×FLAG containing the GFP reporter plasmid. At 48 hpi, GFP fluorescence was determined by flow cytometry. The results are shown as a histogram (left) and as percent relative infectivity (based on mean fluorescence intensity) normalized to the siNC-treated cells (right). Each dot shows the result of an individual experiment. Bars and error bars show mean and standard deviation, respectively. NC, siNC; Rab6a, siRab6a. ****, *P* < 0.0001. (**C**) As in panel **B**, except cells were infected with HPV18 and HPV5 PsV, as indicated. **, *P* < 0.01. (**D**) HeLa cells were transfected with siNC or siRab6a siRNAs and infected with HPV16 PsV harboring the Gluc reporter plasmid at MOI of ~40. At 8 and 16 hpi, proximity ligation assay (PLA) was performed with antibodies recognizing HPV L1 and EEA1. Mock, uninfected; HPV, infected. PLA signals are green; nuclei are blue (4´,6-diamidino-2-phenylindole [DAPI]). Similar results were obtained in two independent experiments. (**E**) The fluorescence of PLA signals was determined from multiple images obtained as in panel **D**. Each dot represents an individual cell (*n* > 40), and black horizontal lines indicate the mean value of the analyzed population in each group. ns, not significant. The graph shows results of a representative experiment. (**F**) As in panel **D**, except PLA was performed at 16 hpi with antibodies recognizing HPV L1 and TGN46. (**G**) Images as in panel **F** were analyzed as described in panel **E**. (**H**) As in panel **D**, except PLA was performed at 16 hpi with antibodies recognizing FLAG (HPV L2) and TGN46. (**I**) Images as in panel **H** were analyzed as described in panel **E**. (**J**) As in panel **D**, except PLA was performed at 24 hpi with antibodies recognizing HPV L1 and GM130. (**K**) Images as in panel **J** were analyzed as described in panel **E**.

We previously reported that Rab6a is required for infection by HPV16 PsV (15), but the specific role of Rab6a in HPV entry is unknown. HPV16 PsV consists of a capsid composed of L1 and FLAG-tagged L2 (i.e., L2 with a 3×FLAG tag appended at its C-terminus) containing a reporter plasmid expressing green fluorescent protein (GFP) or Gaussia luciferase (Gluc). To investigate the role of Rab6a in HPV entry, we assessed the infectivity of HPV16 PsV containing a plasmid expressing GFP in HeLa cells transfected with non-targeting control siRNA (siNC) or siRNA targeting Rab6a expression (siRab6a). Rab6a knockdown was documented by western blotting (Fig. 1A Fig. S1A). Infectivity was measured by flow cytometry for GFP fluorescence at 48 hpi at a multiplicity of infection (MOI) of ~1. This assay measures entry of PsV into the nucleus, resulting in expression of the reporter plasmid. Consistent with our previous report (15), Rab6a knockdown significantly inhibited HPV infectivity (~65%–85% reduction compared to control cells) (Fig. 1B; Fig. S1B). Another siRNA targeting Rab6a had a similar effect on infectivity (Fig. S1A and B). Rab6a depletion also inhibited infection by HPV18 and HPV5 PsVs (Fig. 1C). In addition, Rab6a knockdown inhibited HPV16 PsV infection in human HaCaT skin keratinocytes (Fig. S1C and D). Because normal cell cycle progression is required for HPV entry (32), we tested if Rab6a knockdown affects the cell cycle. Analysis of DNA content in Rab6a knockdown and control HeLa cells revealed no difference, showing that Rab6a depletion does not alter the cell cycle in these cells (Fig. S1E). Collectively, these data demonstrate that Rab6a is required for efficient entry of several pathogenic HPV types.

Rab6a is necessary for cellular protein trafficking from Golgi to ER (33–35). To pinpoint the HPV entry step impaired by Rab6a knockdown, we used proximity ligation assay (PLA), which generates a fluorescent signal in intact cells when two proteins recognized by different antibodies are within 40 nm (36). For these experiments, we infected HeLa cells at the MOI of 40 with HPV16 PsV containing a plasmid expressing Gluc (to avoid interference of GFP fluorescence with the PLA signal). We first examined the proximity of HPV L1 with the endosomal marker protein EEA1. There were negligible L1-EEA1 PLA signals in mock-infected cells. At 8 hpi, similar levels of PLA signals were observed in infected cells transfected with siNC or siRab6a (Fig. 1D and E), indicating that Rab6a is not required for HPV to reach the endosome. At 16 hpi, a time when HPV has largely exited the endosome and entered the TGN in control cells (16, 22), the L1-EEA1 PLA signals were comparable in control and Rab6a knockdown cells (Fig. 1D and E), showing that Rab6a is not required for HPV exit from the endosome.

We then conducted PLA for HPV L1 and marker proteins TGN46 (which is specific for the TGN) or GM130 (which is specific for the *cis*-side of the Golgi stack) (37). At 16 hpi, Rab6a knockdown resulted in much stronger L1-TGN46 PLA signals than in the control cells, indicating that HPV accumulates in TGN in the absence of Rab6a (Fig. 1F and G). Accumulation of HPV in the TGN at 16 hpi in Rab6a-depleted cells was also demonstrated by PLA for L2 and TGN46 (Fig. 1H and I). In contrast, at 24 hpi, infected Rab6a knockdown cells displayed much lower L1-GM130 PLA signals than control cells (Fig. 1J and K), showing that HPV arrival in the *cis-*side of the Golgi stack was impaired in Rab6a-depleted cells. The accumulation of HPV in TGN and its depletion from Golgi stacks caused by Rab6a knockdown demonstrate that Rab6a facilitates HPV entry by supporting HPV trafficking from the TGN to or through the Golgi stack.

### Rab6a is in close proximity to HPV at late times post-infection

**Fig 2:**
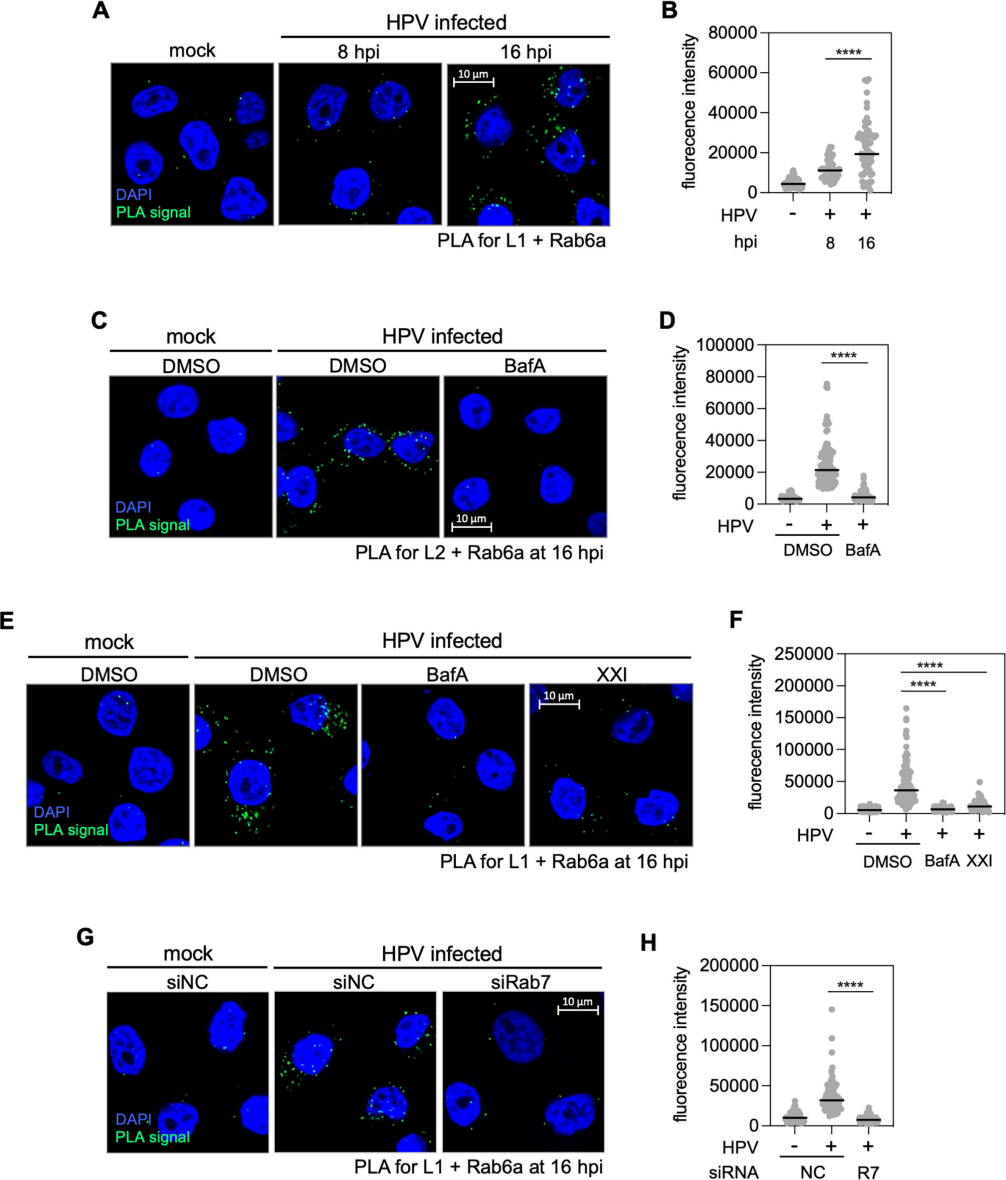
Rab6a engages with HPV at relatively late times during entry. (**A**) HeLa cells were mock-infected or infected at the MOI of ~40 with HPV16 PsV L2-3×FLAG containing the Gluc reporter plasmid. At 8 and 16 hpi, PLA was performed with antibodies recognizing HPV L1 and Rab6a. PLA signals are green; nuclei are blue. Similar results were obtained in two independent experiments. (**B**) The fluorescence of PLA signals was determined from multiple images obtained as in panel **A**. Each dot represents an individual cell (*n* > 40), and black horizontal lines indicate the mean value of the analyzed population in each group. ****, *P* < 0.0001. The graph shows results of a representative experiment. (**C**) As in panel **A**, except dimethylsulfoxide (DMSO) or bafilomycin A1 (BafA) were added to the medium 30 min prior to infection, and at 16 hpi, PLA was performed with antibodies recognizing FLAG (HPV L2) and Rab6a. (**D**) Images as in panel **C** were analyzed as described in panel **B**. (**E**) As in panel **A**, except DMSO, BafA, or γ-secretase inhibitor XXI was added to the medium 30 min prior to infection, and at 16 hpi, PLA was performed with antibodies recognizing HPV L1 and Rab6a (**F**) Images as in panel **E** were analyzed as described in panel **B**. (**G**) HeLa cells were transfected with negative control (siNC) or Rab7-targeting siRNA (siRab7) and infected at the MOI of ~40 with HPV16 PsV L2-3×FLAG containing the Gluc reporter plasmid. At 16 hpi, PLA was performed with antibodies recognizing HPV L1 and Rab6a. Similar results were obtained in two independent experiments. (**H**) Images as in panel **G** were analyzed as described in panel **B**. R7, siRab7.

If Rab6a acts directly on HPV during entry, we predict that Rab6a and HPV will be in close proximity when the virus is in the TGN. We conducted additional PLA experiments to determine if this is the case. Minimal L1-Rab6a PLA signals were detected in uninfected cells, and only modest signals were detected at 8 hpi (Fig. 2A and B). In contrast, at 16 hpi, we detected strong L1-Rab6a PLA signals (Fig. 2A and B) and strong L2-Rab6a PLA signals (Fig. 2C and D). Thus, Rab6a is in proximity to HPV at 16 hpi, but not at 8 hpi (Fig. 2A through D), consistent with its role in HPV trafficking out of the TGN.

Because Rab6a is in proximity to HPV primarily at later stages of entry and is required for exit of HPV from the TGN, we predicted that blocking transport of HPV to the TGN would prevent the HPV-Rab6a interaction. To test this, we used PLA at 16 hpi to assess the association of HPV and Rab6a in cells where HPV trafficking out of the endosome was blocked. Endosomal acidification, γ-secretase activity, and Rab7 are required for HPV trafficking from endosome to TGN at early stages of infection (10, 20, 38, 39). Rab6a association with L1 as assessed by PLA at 16 hpi was markedly reduced by treatment with bafilomycin A1 (BafA, an inhibitor of endosome acidification), XXI (an inhibitor of γ-secretase), or by Rab7 knockdown (Fig. 2E through H). Similarly, L2-Rab6a PLA signal at 16 hpi was abolished by BafA (Fig. 2C and D). The decreased PLA signal due to BafA or XXI treatment was not due to redistribution of Rab6a away from the TGN, as assessed by Rab6a-TGN46 PLA (Fig. S2). Collectively, these findings show that endosome acidification and the action of Rab7 and γ-secretase are required for HPV to come into proximity with Rab6a. We conclude that arrival of HPV in the TGN is necessary for HPV-Rab6a association and that the low L1-Rab6a PLA signal at 8 hpi reflects the leading edge of HPV arrival in the TGN.

### Rab6a is required for HPV-BICD2-dynein interaction in the TGN but not in the endosome

**Fig 3:**
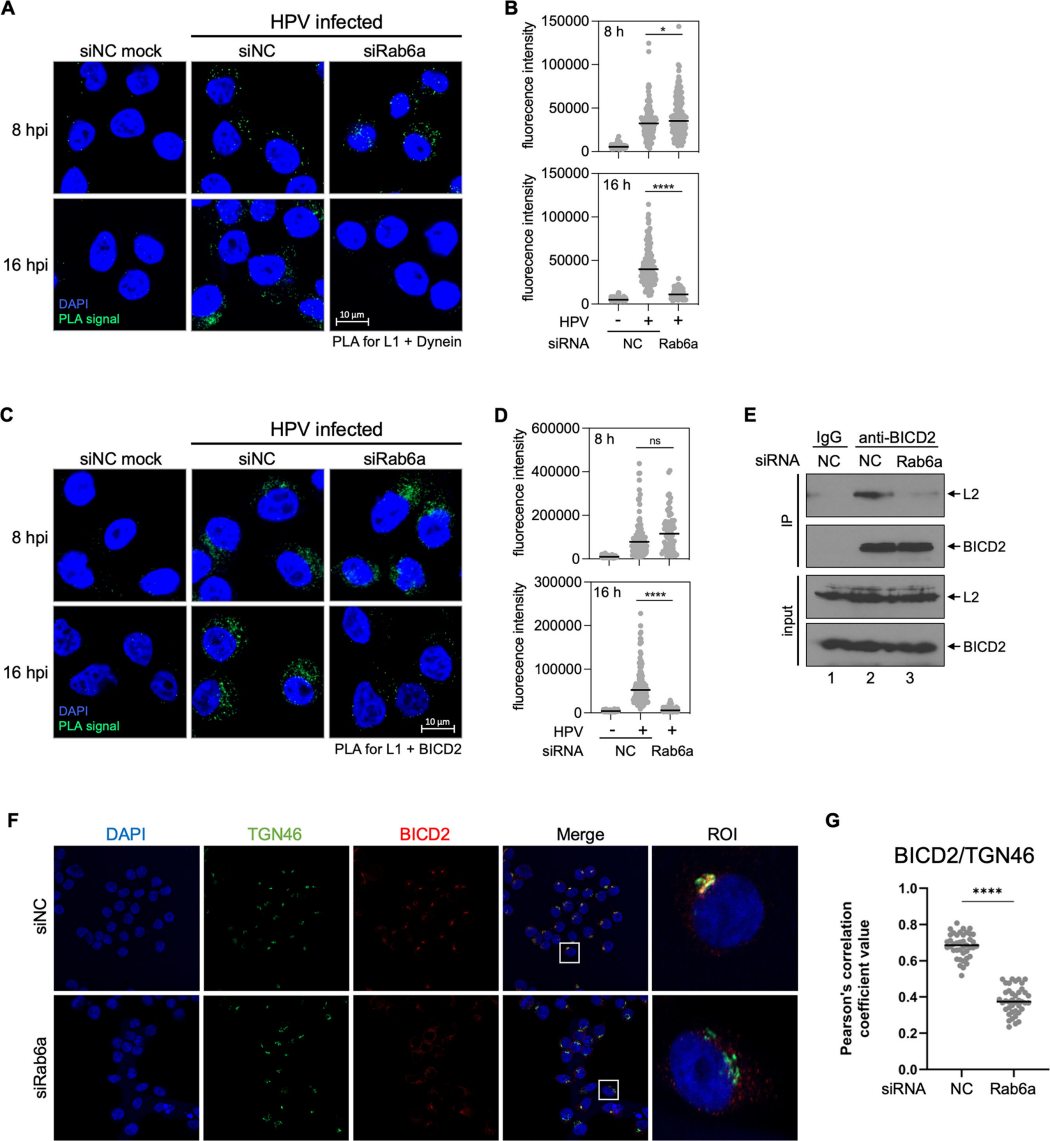
Rab6a is required for HPV-BICD2-dynein association in the TGN. (**A**) HeLa cells were transfected with siNC or siRab6a siRNAs and infected with HPV16 PsV containing the Gluc reporter plasmid at the MOI of ~40. At 8 and 16 hpi, PLA was performed with antibodies recognizing HPV L1 and dynein. Mock, uninfected; HPV, infected. PLA signals are green; nuclei are blue. Similar results were obtained in two independent experiments. (**B**) The fluorescence of PLA signals was determined from multiple images obtained as in panel **A**. Each dot represents an individual cell (*n* > 40), and black horizontal lines indicate the mean value of the analyzed population in each group. *, *P* < 0.05; ****, *P* < 0.0001. The graph shows results of a representative experiment. (**C**) As in panel **A**, except PLA was performed with antibodies recognizing HPV L1 and BICD2. (**D**) Images as in panel **C** were analyzed as described in panel **B**. (**E**) siRNA-transfected HeLa cells were infected with HPV harboring the GFP reporter plasmid at the MOI of ~1. At 16 hpi, cells were lysed and immunoprecipitated with control antibody (IgG) or antibody recognizing BICD2. Precipitated samples were subjected to western blot analysis using an antibody recognizing FLAG (HPV L2) or BICD2. (**F**) HeLa cells were transfected with siNC or siRab6a. After 48 h, BICD2 and TGN46 were stained using antibodies recognizing BICD2 and TGN46. Immunofluorescence (IF) images were shown; TGN46, green; BICD2, red; nuclei, blue. Merged image shows TGN46 and BICD2 with overlap were pseudocolored yellow. ROI, region of interest. Similar results were obtained in three independent experiments. (**G**) Pearson’s correlation coefficient values for TGN46 and BICD2 colocalization in multiple images obtained as in panel **F**. Each dot represents an individual cell (*n* > 40), and horizontal lines indicate the mean value of the analyzed population in each group. ****, *P* < 0.0001. The graph shows results of a representative experiment.

Dynein is known to interact with Rab6a and L2 and is thought to play a role in retrograde trafficking of HPV during entry (25, 28, 40, 41). We used PLA for L1 and dynein at 8 and 16 hpi to test whether Rab6a is required for HPV-dynein association in infected HeLa cells. In control cells, clear L1-dynein PLA signals were detected at both 8 and 16 hpi but were negligible in mock-infected cells (Fig. 3A and B), indicating that HPV associates with dynein during entry, consistent with previous reports (25, 28, 29). Notably, at 16 hpi but not at 8 hpi, HPV-dynein interaction was markedly decreased in cells depleted of Rab6a. These results indicate that Rab6a plays a critical role in HPV-dynein association at 16 hpi when the virus is primarily in the TGN, but it is not required for HPV-dynein interaction at 8 hpi when the virus is primarily in the endosome.

We recently reported that BICD2, a cargo adaptor for dynein, binds HPV L2 and is required for HPV-dynein association during HPV entry (30, 42, 43). Furthermore, it is well-established that Rab6a interacts with BICD2 and promotes dynein-mediated cargo trafficking (43–46). Thus, we hypothesized that Rab6a may enable the HPV-BICD2 interaction in the TGN and thereby promote HPV-dynein association. We used PLA for L1 and BICD2 at 8 and 16 hpi to test whether Rab6a knockdown affects HPV interaction with BICD2 in infected HeLa cells. At both 8 and 16 hpi, L1-BICD2 PLA signals were readily detected in control cells (Fig. 3C and D). At 8 hpi, comparable L1-BICD2 signals were detected in control and Rab6a knockdown cells. In contrast, at 16 hpi, L1-BICD2 PLA signals were greatly reduced by Rab6a knockdown, although Rab6a knockdown did not affect expression of BICD2 at this timepoint (Fig. 3E). These results indicate that Rab6a is required for HPV-BICD2 interaction in the TGN at 16 hpi, but not at 8 hpi, when the incoming virus is predominantly in the endosome. We note that BICD2 localization at the TGN was reduced by Rab6a knockdown in uninfected cells (Fig. 3F and G), a finding that presumably explains at least in part the decreased HPV-BICD2 PLA signal in Rab6a-depleted cells.

As an independent approach to assess the interaction of HPV and BICD2, we performed co-immunoprecipitation experiments from detergent lysates of control and Rab6a knockdown HeLa cells at 16 hpi. The anti-BICD2 antibody, but not the control IgG, co-immunoprecipitated (co-IPed) the L2 protein from control infected cells (Fig. 3E, lanes 1 and 2) as expected, but the amount of L2 protein co-IPed with BICD2 was markedly reduced from lysates of Rab6a knockdown cells (Fig. 3E, lanes 2 and 3). Taken together with the PLA experiments described above, these results indicate that Rab6a is required for optimal HPV-BICD2 interaction in intact cells and cell extracts at 16 hpi and imply that Rab6a facilitates HPV-BICD2-dynein association at the TGN but not at earlier steps in entry.

### Excess GTP- or GDP-bound Rab6a impairs HPV entry

**Fig 4:**
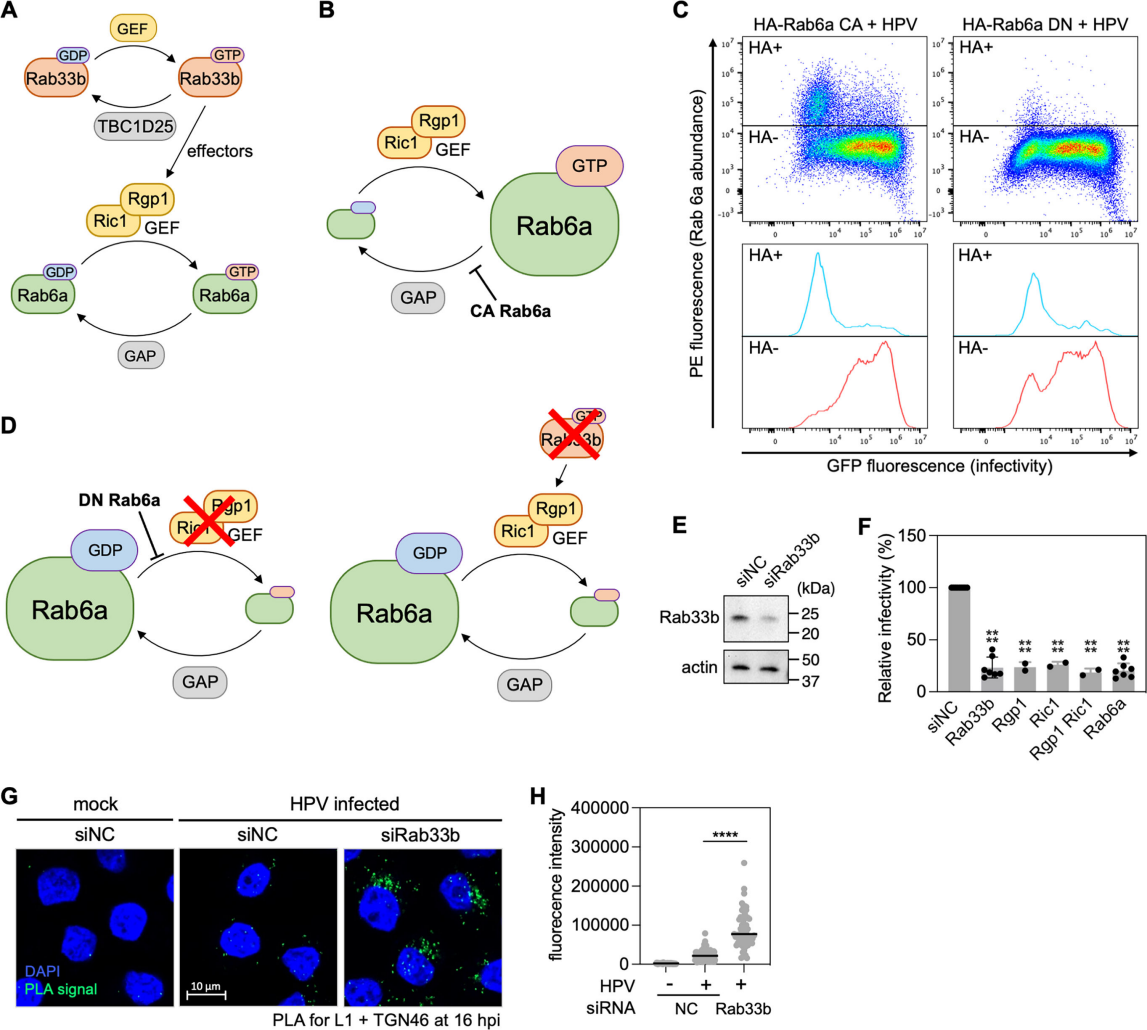
Excess of either GTP-Rab6a or GDP-Rab6a impairs HPV entry. (**A**) Schematic of Rab6a cycling between GTP-bound and GDP-bound forms. Ric1/Rgp1, a Rab6a guanine nucleotide exchange factor (GEF) complex, converts GDP-Rab6a to GTP-Rab6a by exchanging GTP for GDP. A GTPase-activating protein (GAP) hydrolyzing GTP-Rab6a is yet to be identified. Ric1 and Rgp1 are effectors of Rab33b. TBC1D25 is a GAP for Rab33b, and a guanine nucleoside exchange factor (GEP) for Rab33b has not been identified. (**B**) CA Rab6a mutant is locked in GTP-bound form; thus, cells expressing CA Rab6a accumulate GTP-Rab6a. (**C**) 293TT cells were transfected with a plasmid expressing HA-tagged CA or DN Rab6a, as indicated. At 48 h after transfection, cells were infected at the MOI of ~1 with HPV16 PsV L2-3×FLAG containing the GFP reporter plasmid. At 48 hpi, samples were stained using phycoerythrin (PE)-conjugated antibody recognizing HA, and flow cytometry was used to determine PE and GFP fluorescence. Cells expressing HA-tagged CA or DN Rab6a were marked as HA+, and those not expressing these proteins were marked as HA−. Representative dot plots of individual cells are shown in top panels. Corresponding histograms are shown on the bottom. (D) Dominant negative (DN) Rab6a mutant is locked in GDP-bound form; thus, cells expressing DN Rab6a accumulate GDP-Rab6a. Knockdown of Ric1/Rgp1 or Rab33b also results in GDP-Rab6a accumulation. (**E**) HeLa cells were transfected with negative control (siNC) or Rab33b-targeting siRNA (siRab33b) and subjected to western blot analysis using an antibody recognizing Rab33b (top panel) and actin (bottom panel). (**F**) siRNA-treated HeLa cells were infected at the MOI of ~1 with HPV16 PsV L2-3×FLAG containing the GFP reporter plasmid. At 48 hpi, GFP fluorescence was determined by flow cytometry, and infectivity relative to control was calculated. Each dot shows the result of an individual experiment. Bars and error bars show mean and standard deviation, respectively. ****, *P* < 0.0001. (**G**) HeLa cells were transfected with siNC or siRNAs targeting Rab33b. At 40 h post transfection, cells were mock-infected or infected at the MOI of ~40 with HPV16 PsV L2-3×FLAG containing the Gluc reporter plasmid. At 16 hpi, PLA was performed with antibodies recognizing HPV L1 and TGN46. Mock, uninfected; HPV, infected. PLA signals are green; nuclei are blue. Similar results were obtained in two independent experiments. (**H**) The fluorescence of PLA signals was determined from multiple images obtained as in panel **G**. Each dot represents an individual cell (*n* > 40), and black horizontal lines indicate the mean value of the analyzed population in each group. ****, *P* < 0.0001. The graph shows results of a representative experiment.

Because the GTP-bound form of Rab proteins typically promotes trafficking of cellular protein cargo (1), we assumed that GTP-Rab6a promotes HPV entry. To test this, we introduced constitutively active (CA) HA-tagged Rab6a mutant (Q72L) into 293TT cells to induce a higher GTP-Rab6a to GDP-Rab6a ratio compared to normal cells (Fig. 4A and B) (33, 40, 47). Forty-eight hours after transfection with a plasmid expressing CA Rab6a, cells were infected with HPV16 PsV. At 48 hpi, we used flow cytometry to measure GFP fluorescence to determine HPV infection and anti-HA staining to measure expression of the mutant Rab6a protein. This experimental design allowed us to compare HPV entry in the presence or absence of CA Rab6a (i.e., in HA-positive and HA-negative cells, respectively) within the same cell population. This design was essential, because a relatively small number of cells expressed exogenous Rab6a (Fig. 4C). Contrary to our expectation, cells expressing CA Rab6a showed reduced HPV infection compared to cells lacking CA Rab6a (Fig. 4C, left). This result indicates that excess GTP-Rab6a impairs HPV infection.

Next, we investigated the effect of excess GDP-Rab6a by transiently expressing the dominant negative (DN) Rab6a mutant (T27N), which is predicted to cause accumulation of GDP-bound Rab6a (Fig. 4A and D, left) (33, 47). Expression of HA-tagged DN Rab6a also inhibited HPV infection in 293TT cells compared to cells lacking DN Rab6a (Fig. 4C, right), indicating that excess GDP-Rab6a also impairs HPV infection. As an alternative approach to generate HeLa cells with excess GDP-Rab6a, we knocked down Rgp1 and Ric1, which form a complex that displays guanine nucleotide exchange factor (GEF) activity for Rab6a to generate GTP-Rab6a (Fig. 4D, left) (48, 49). Thus, Rgp1/Ric1 knockdown is predicted to result in increased GDP-Rab6 (Fig. 4D, left). Individual knockdown of Rgp1 or Ric1, as well as combined knockdown of both proteins, reduced HPV infection (Fig. 4F), supporting the conclusion that excess GDP-Rab6a impairs HPV infection. Finally, we knocked down Rab33b (Fig. 4E), which recruits Rgp1/Ric1 GEF to Rab6a and is thus also predicted to increase GDP-Rab6a (Fig. 4D, right) (48), and tested infectivity and localization of the incoming HPV. Rab33b knockdown also inhibited HPV infection (Fig. 4F) and resulted in HPV accumulation at TGN (Fig. 4G and H). Taken together, these results show that excess of either GTP-bound or GDP-bound Rab6a impairs HPV entry.

### A C-terminal segment of HPV L2 preferentially interacts with GTP-bound Rab6a *in vitro*

**Fig 5:**
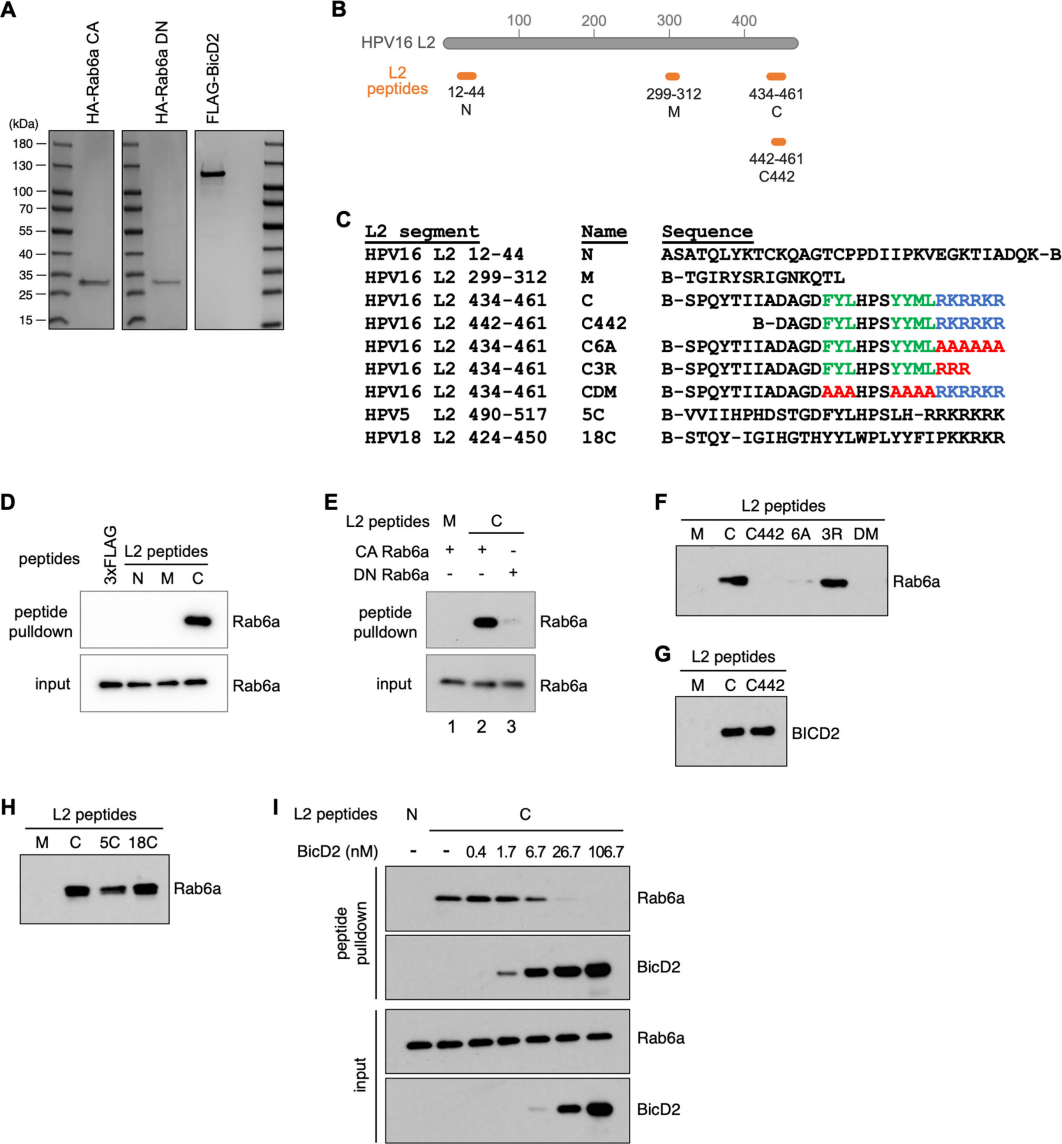
C-terminal segment of L2 preferentially interacts with GTP-Rab6a. (**A**) Purified HA-tagged CA and DN Rab6a and FLAG-tagged BICD2 proteins were separated in SDS-PAGE and visualized by Coomassie blue staining. Molecular weights of size markers are listed on the left. (**B**) Schematic of HPV16 L2 (red) and L2 peptides covering different segments in L2 (orange). Numbers under each peptide indicate amino acid positions from full-length HPV16 L2. Name of each peptide is indicated under those numbers. (**C**) Sequences of the HPV L2 peptides used in this study. B Indicates biotin. Amino acids of the HPV16 retromer binding site are shown in green, and those of the CPP are shown in blue. (**D**) Purified HA-tagged CA Rab6a protein was incubated with indicated biotinylated L2 peptides. Peptide-associated proteins were then pulled down using streptavidin beads and subjected to western blot analysis using an antibody recognizing HA. Similar results were obtained in three independent experiments. Data from a representative experiment are shown. (**E**) As in panel **D**, except either CA or DN Rab6a protein was used. (**F and G**) As in panel **D**, except purified HA-tagged CA Rab6a (**F**) or FLAG-tagged BICD2 (**G**) was used. (**H**) As in panel **D**, except using indicated biotinylated L2 peptides (M, C, 5C from HPV5 L2, or 18C from HPV18 L2). (**I**) Purified HA-tagged CA Rab6a (3.4 nM) or FLAG-tagged BICD2 (at indicated concentrations) was incubated with indicated biotinylated HPV16 L2 peptide (N or C). Peptide-associated proteins were then pulled down using streptavidin beads and subjected to western blot analysis using an antibody recognizing FLAG or HA. Similar results were obtained in two independent experiments. Data from a representative experiment are shown.

To determine if Rab6a can bind directly to L2 and to identify which portion of L2 interacts with Rab6a, we conducted *in vitro* pull-down experiments using purified HA-tagged CA Rab6a (Fig. 5A) and biotinylated peptides corresponding to amino acids 12–44 (designed L2 N in the figure), 299–312 (L2 M), or 434–461 (L2 C) of HPV16 L2 (Fig. 5B and C). Each L2 peptide or 3×FLAG peptide as negative control was incubated with purified CA Rab6a protein, then the peptides and any associated Rab6a were pulled down using streptavidin beads and Rab6a was detected by immunoblotting. Of these four different peptides, only peptide 434–461 pulled down CA Rab6a protein (Fig. 5D). This result indicates that the C-terminus portion of L2 (434–461) can interact directly with Rab6a.

We also purified HA-tagged DN Rab6a protein (Fig. 5A) and performed pull-down experiments using the C-terminal L2 peptide and CA or DN Rab6a. As described above, peptide 434–461 pulled down CA Rab6a, whereas peptide 299–312 did not (Fig. 5E, lanes 1 and 2). In contrast, peptide 434–461 pulled down very little DN Rab6a (Fig. 5E, lanes 2 and 3), indicating that L2 preferentially binds to GTP-Rab6a *in vitro* compared to GDP-Rab6a.

We next used mutant L2 C peptides to test whether the highly conserved sequence elements in L2 C, namely the retromer binding site (RBS) and the CPP, are necessary for L2 C binding to Rab6a (Fig. 5C). Substitution of either the RBS or the CPP with alanines (to generate peptides CDM and C6A, respectively) markedly impaired binding to Rab6a (Fig. 5F), indicating that Rab6a binding to the L2 C terminus requires both the RBS and the CPP. We previously reported that these alanine mutations interfered with BICD2 binding (30). However, replacing the CPP with three arginines (C3R), which severely inhibits cell-penetrating function (20), did not inhibit L2 C binding to Rab6a, suggesting that a basic region rather than an active CPP downstream of the RBS is required to support L2-Rab6a binding (Fig. 5F).

### Overlapping segments of HPV L2 are required for *in vitro* interaction with Rab6a and BICD2

Although BICD2 association with HPV requires Rab6a in infected cells at 16 hpi (Fig. 3C and D), we previously reported *in vitro* binding experiments showing that BICD2 can bind directly to L2 in the absence of Rab6a (30). Here, we first used the L2 C peptide to confirm that Rab6a and BICD2 can bind independently to the same segment of L2 *in vitro*. When tested individually, purified FLAG-tagged BICD2 and HA-tagged CA Rab6a were both pulled down by peptide L2 C but not by peptide L2 M (Fig. 5F and G). Thus, *in vitro*, BICD2 was able to bind this L2 segment in the absence of Rab6a, consistent with our previous report (30).

We then tested binding using a shorter HPV16 L2 peptide 442–461 (designated C442) (Fig. 5B and C). Interestingly, this 20-amino acid-long peptide precipitated as much BICD2 as did peptide 434–461, as reported previously (30), but it did not pull down Rab6a (Fig. 5F and G). This result indicates that, although BICD2 and Rab6a both bind *in vitro* to a peptide containing a C-terminal segment of L2, removal of the N-terminal eight amino acids of the peptide inhibits Rab6a binding but not BICD2 binding.

Although the RBS and CPP are highly conserved in papillomavirus L2 proteins, the amino acid sequence that is missing from HPV16 L2 peptide C442 is not conserved (Fig. 5C and S3). To determine whether the L2 C terminus of other HPV types binds Rab6a, we performed *in vitro* pull-down experiments with C-terminal peptides derived from HPV18 and HPV5 L2, designated 18C and 5C, respectively (Fig. 5C). As shown in Fig. 5H, the HPV18 and HPV5 L2 peptides also bound Rab6a, albeit to a lesser extent for 5C (from HPV5) compared to the HPV16 L2 C peptide, even though they lack sequences homologous to the upstream sequences that are required for the HPV16 peptide to bind Rab6a. Thus, the C-terminal segment of L2 from diverse pathogenic HPV types binds Rab6a, consistent with the Rab6a requirement for these viruses to infect cells (Fig. 1B and C). Furthermore, although removal of HPV16 L2 positions 434 to 441 blocks binding of the HPV16 L2 peptide to Rab6a, the ability of the HPV5 and HPV18 L2 peptides to bind Rab6a suggests that the precise sequence of this upstream segment is not required.

Because the binding sites for BICD2 and Rab6a on L2 C overlap, it is possible that BICD2 can displace Rab6a from binding to L2. To test this, we performed *in vitro* pull-down experiments by incubating the L2 C peptide with a fixed amount of Rab6a and a range of BICD2 concentrations (Fig. 5I). As shown in Fig. 5I, top panel, increasing BICD2 amounts caused a decrease in the amount of Rab6a pulled down by the peptide, indicating that BICD2 and Rab6a compete for binding the L2 C peptide.

## DISCUSSION

**Fig 6:**
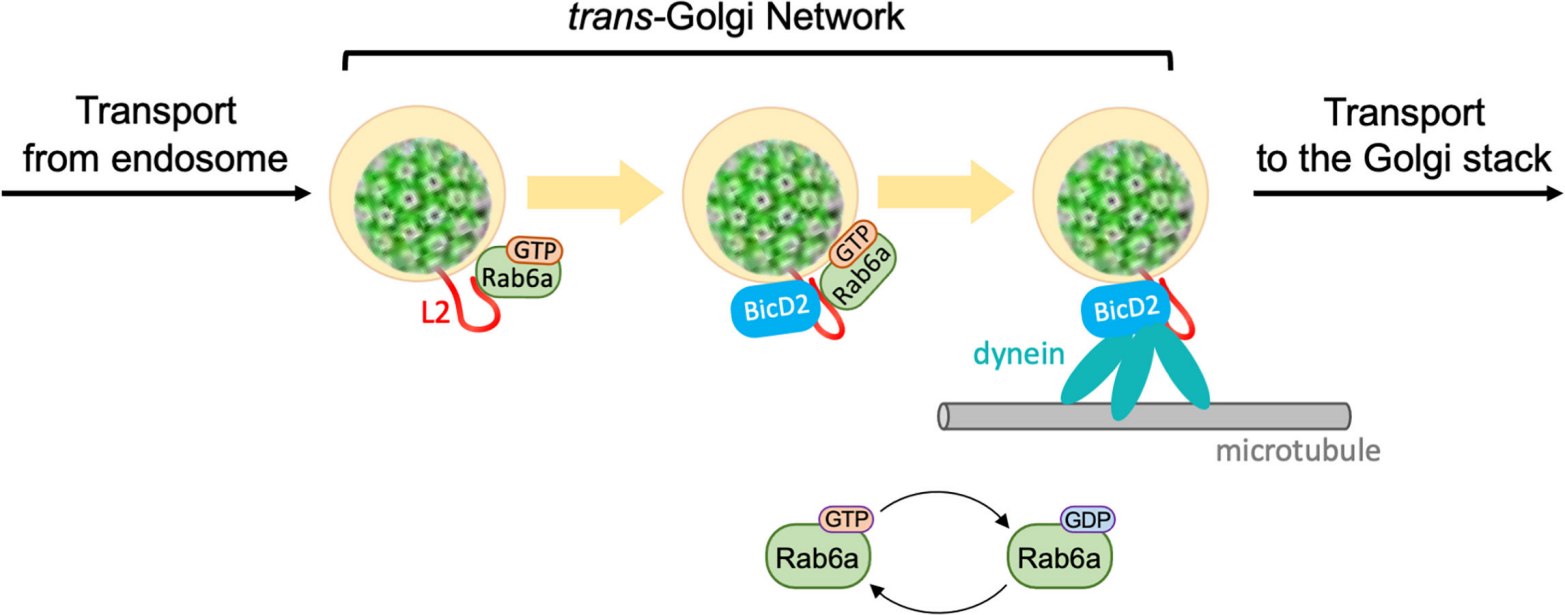
Model for the role of Rab6a in promoting HPV trafficking from the *trans*-Golgi network to the Golgi stack. After retrograde trafficking of internalized HPV to *trans-*Golgi network, L2 associates with GTP-Rab6a, which recruits BICD2, an adaptor for dynein complex. Hydrolysis of GTP-Rab6a and/or BICD2 binding promotes dissociation of Rab6a from L2, leaving L2 associated with BICD2/dynein, which mediates HPV cargo trafficking on microtubules to the Golgi stack.

Rab GTPases play pivotal roles in HPV entry (1–3, 6). Here, we used multiple approaches to confirm the importance of Rab6a for HPV infection, including knockdown of Rab6a itself, expression of Rab6a mutants, knockdown of proteins regulating Rab6a activity, and imaging and biochemical analysis of the association of HPV and Rab6a and the consequences of this association. Our findings reveal that upon arrival at the TGN, Rab6a associates with HPV and promotes the assembly or maintenance of an HPV-BICD2-dynein complex, thereby allowing dynein-mediated TGN exit and intra-Golgi HPV trafficking (Fig. 6). Rab6a is required for efficient infection for HPV16, HPV18, and HPV5, and the C-terminus of L2 of all three HPV types can bind purified Rab6a. These results suggest that direct binding of Rab6a to the C-terminus of L2 supports exit of a variety of pathogenic HPV types from TGN. We previously reported that retromer/L2 binding mediates endosome exit, and COPI/L2 binding supports HPV transit through the TGN/Golgi (22, 27). Thus, HPV entry is mediated by an intricate retrograde trafficking mechanism that sequentially employs multiple different host factors.

Rab6a depletion abrogates HPV-BICD2 and HPV-dynein association in the TGN in HPV-infected cells as assessed by PLA and co-IP at 16 hpi. Thus, HPV-BICD2 association in the TGN in infected cells requires Rab6a. This is consistent with Rab6a localization to the Golgi fraction or the TGN and Golgi stack as assessed by proteomics and imaging studies (50, 51). Our results also imply that the L2-dynein association previously detected by co-IP from HPV-infected cell lysates may be mediated by endogenous Rab6a and/or BICD2 (25, 28, 29).

Rab proteins exist in two forms: an active GTP-bound state necessary for interacting with effector proteins that supports the trafficking of cellular cargo and an inactive GDP-bound state (1, 3). Typically, excess GTP-Rab6a allows the transport of cellular cargo proteins, while excess GDP-Rab6a blocks transport (33, 34). Consistent with this paradigm, L2 preferentially associates with GTP-Rab6a compared to GDP-Rab6a (Fig. 5E). Unlike typical cellular cargo trafficking, however, excess of either the GTP- or the GDP-bound form of Rab6a impairs HPV entry (Fig. 4D). Similarly, excess of either GTP-Rab7 or GDP-Rab7 inhibits HPV entry (10, 11) and excess GDP-Rab9a promotes HPV entry while GTP-Rab9a inhibits it (11). Furthermore, Rab6a promotes retrograde trafficking of cellular cargo from the *cis*-Golgi to the ER (33, 34) as well as anterograde intra-Golgi trafficking (47, 52, 53). In contrast, our results show that Rab6a supports retrograde HPV trafficking from the TGN to the Golgi stack, suggesting that Rab6a mediates a different step of retrograde trafficking for HPV compared to cellular protein cargo. Taken together, these results indicate that HPV may in effect reprogram Rab proteins and associated cellular trafficking machinery to support virus entry, implying that there is considerable plasticity of Rab proteins in supporting a variety of trafficking events.

Rab proteins function at various stages during HPV entry. Once HPV L2 protrudes across the endosomal membrane, it sequentially interacts with Rab9a and later Rab7, which support HPV trafficking from endosome to TGN by regulating HPV-retromer association and dissociation (10, 11). After arrival at the TGN, HPV L2 associates with GTP-Rab6a, which recruits BICD2 and dynein, thereby assembling a complex that mediates trafficking of HPV from the TGN (Fig. 6). Consistent with this sequential model, Rab6a is not required for the early steps of entry prior to TGN entry, and HPV-Rab6a association requires endosome acidification, functional γ-secretase, and Rab7, all of which must occur for HPV to exit the endosome. The preferential binding of the GTP-bound form of Rab6a to both L2 and BICD2 (Fig. 5E) (43–45) may facilitate the recruitment of BICD2 to L2.

We previously reported that BICD2 knockdown results in HPV accumulation in endosomes and the TGN (30), and we show here that HPV-dynein association and HPV-BICD2 association is independent of Rab6a at 8 hpi (Fig. 3A through D). In contrast, at 16 hpi Rab6a knockdown disrupts or prevents the HPV-dynein and HPV-BICD2 interaction in the TGN and causes accumulation in the TGN but not in the endosome. We conclude that BICD2 and dynein are recruited to HPV in the endosome in a Rab6a-independent manner, possibly by as-yet-unidentified factor(s) such as another Rab protein, but once HPV enters the TGN, Rab6a is required to stabilize, maintain, or reinstate the HPV-BICD2 interaction to allow further dynein-mediated transport of the incoming virus. Because Rab6a knockdown blocks TGN exit of HPV, we cannot determine if Rab6a also acts at more downstream steps of entry, such as transit across the Golgi stack or Golgi-to-ER trafficking, the latter a step Rab6a can mediate for cellular cargo.

HPV localizes to the *cis*-Golgi GM130 compartment at late times during entry, and HPV infection is inhibited by brefeldin A, which disrupts the Golgi stack (17, 37, 54). These results suggest that incoming HPV must transit across the stack to successfully infect cells. It is likely that HPV trafficking from the TGN and through the Golgi stacks involves iterative rounds of vesicle budding, transport, and fusion, and Rab6a may act repetitively during this process. Indeed, the reduced binding of GDP-Rab6a to L2 may facilitate the dissociation of Rab6a from L2 upon GTP hydrolysis, only to reassociate at a later step when GTP-Rab6a is regenerated, providing an explanation for the apparent requirement for Rab6a cycling for HPV transport.

Although Rab6a is required for HPV-BICD2 association in the TGN of infected cells, a C-terminal segment of L2 can directly interact with purified BICD2 *in vitro*, indicating that L2 can bind BICD2 independently of Rab6a. The ability of peptide C442 (positions 442–461) to bind BICD2 but not Rab6a confirms that BICD2 can bind L2 independently of Rab6a. Direct binding of BICD2 to L2 could play a role in assembling the complex prior to the Rab6a-dependent step, and/or it could stabilize the complex between HPV, BICD2, and dynein in the TGN. Our peptide pull-down studies show that Rab6a and BICD2 have overlapping but distinct sequence requirements for binding to L2, and that they can compete for L2 binding. Rab6a-mediated recruitment of BICD2 to L2 may help displace Rab6a so it can bind to another L2 molecule.

The sequence corresponding to HPV16 434–441 is divergent in HPV5 and HPV18 L2, yet peptides from these HPV types bind Rab6a, implying that these sequences are not specifically required for Rab6a binding. We note that the more divergent HPV5 L2 peptide displayed slightly reduced binding to Rab6a compared to the peptide from HPV16 or HPV18, suggesting that the amino acid sequence upstream of position 442 influences L2-Rab6a binding. Alternatively, binding of Rab6a but not BICD2 may simply require additional amino acids to position the core binding site farther from the N-terminus of the peptide.

In addition to BICD2 and Rab6a, the C-terminal segment of HPV16 L2 also binds retromer, so presumably, the binding of various factors to this region of L2 is precisely orchestrated to allow proper trafficking. The L2 mutations that prevent BICD2 or Rab6a binding also directly interfere with CPP function or prevent retromer binding, which is required for stable L2 protrusion into the cytoplasm (55). Therefore, we cannot use these mutations to assess the role of BICD2 or Rab6a during entry because they prevent L2 protrusion, a step required for BICD2 and Rab6a binding in infected cells.

Rab6a also plays important roles in infection by other viruses. Rab6a knockdown impairs herpes simplex virus type 1 (HSV1) infection by inhibiting HSV1 glycoprotein anterograde trafficking from Golgi to the plasma membrane and capsid envelopment (56). In addition, Rab6 promotes production of infectious human cytomegalovirus by supporting the trafficking of viral protein(s) into the viral assembly compartment (57). HIV infection also requires Rab6a, although the specific step Rab6a mediates is not known (58). Thus, different viruses appear to employ Rab6a for different functions during virus entry, assembly and/or, exit.

Our study elucidates a role of Rab6a in facilitating HPV entry by promoting HPV-BICD2-dynein association in the TGN, enabling retrograde trafficking of HPV from the TGN to the Golgi stack. Rab6a engages with HPV by interacting with L2 after the incoming virus has arrived at the TGN. This function of Rab6a not only sheds light on virus entry mechanisms but also provides insights into cellular protein trafficking, albeit with distinct Rab6a functions compared to known cellular cargo. Our findings also reveal sequential interactions between viral and host proteins during HPV entry, potentially identifying targets for therapeutic interventions to inhibit infection.

## MATERIALS AND METHODS

### Cell lines

HeLa S3 cells (herein HeLa cells) were purchased from American Type Culture Collection (ATCC). HaCaT cells were purchased from AddexBio Technologies. 293TT cells were generated by introducing SV40 large T antigen cDNA into HEK293T cells to increase large T antigen expression and were obtained from Christopher Buck (NIH). Cell lines were verified by using the ATCC cell authentication service. All cell lines were cultured at 37°C and 5% CO_2_ in Dulbecco’s modified Eagle’s medium (DMEM) supplemented with 20 mM HEPES, 10% fetal bovine serum, L-glutamine, and 100 units/mL penicillin streptomycin (DMEM10).

### Production and titering of HPV PsV

HPV16 PsVs were produced by co-transfecting 293TT cells with wild-type p16SheLL-3×FLAG tag (16) together with pCINeo-GFP (obtained from C. Buck) or pCINeo-Gluc (27) using polyethyleneimine (MilliporeSigma). For HPV18 and HPV5, p18SheLL-HA tag and p5SheLL were used, respectively. PsVs were purified by density gradient centrifugation in OptiPrep (MilliporeSigma) as described (59). Briefly, cells were washed with Dulbecco’s phosphate buffered saline (DPBS) at 24 h post transfection, incubated in DMEM10 for an additional 48 h, and collected in siliconized tubes. The cells were then incubated in lysis buffer (DPBS with 0.5% Triton X-100, 10 mM MgCl_2_, 5 mM CaCl_2_, 100 µg/mL RNase A [Qiagen]) overnight at 37°C to allow capsid maturation. The lysates containing matured PsVs were loaded on an OptiPrep gradient that had been stabilized at least 1 h and centrifuged at 50,000 × *g* for 3.5 h–4 h at 4°C in a SW-55Ti rotor (Beckman). Fractions were collected in siliconized tubes and subjected to SDS-PAGE followed by Coomassie blue staining to assess the abundance of L1 and L2 proteins. Peak fractions were pooled, aliquoted, and stored at −80°C.

PsV titer was determined in HeLa cells infected with serial dilutions of PsV. The percentage of cells expressing GFP was determined by flow cytometry. MOI of 1 corresponds to the concentration of PsV that results in 60%–70% of HeLa cells expressing GFP.

### Determining HPV PsV infectivity

HeLa cells (0.37 × 10^5^ cells per well) were plated in 24-well plates ~48 h prior to infection. Approximately 6 h later, cells were transfected with 6.7 nM of indicated siRNAs (Table S1) using Lipofectamine RNAiMAX (Invitrogen) according to manufacturer’s protocol. Non-targeting siRNA was used as a negative control. Forty to forty-eight hours after transfection, cells were infected with PsVs at an infectious MOI of ~1. At 48 hpi, infectivity was determined by using flow cytometry to measure fluorescence intensity produced from expression of the reporter gene. Relative percent infectivity was determined by normalizing mean fluorescence intensity of samples transfected with experimental siRNA to that of the cells transfected with control siRNA, which was set at 100%. To measure infectivity in HaCaT cells, they were infected with four to five times the amount of PsVs used to measure infectivity in HeLa cells (resulting in infection of ~2/3 of the cells).

### Western blot analysis

siRNA-treated cells were lysed using ice-cold 1× radioimmunoprecipitation assay (RIPA) buffer (50 mM Tris [pH 7.4], 150 mM NaCl, 1% Nonidet P-40, 1% sodium deoxycholate, 0.1% sodium dodecyl sulfate, 1 mM ethylenediaminetetraacetic acid) supplemented with 1× HALT protease inhibitor cocktail (Pierce) for 15 min at 4°C. After centrifugation at 14,000 rpm for 15 min at 4°C, the protein concentration in the supernatant was determined by bicinchoninic acid (BCA) protein assay (Pierce). After normalization for protein amounts, the supernatant was mixed with 4× Laemmli dye (Bio-Rad) supplemented with 10% 2-mercaptoethanol and incubated in a water bath for 7 min at 100°C. For pull-down experiments, samples were prepared as described below. Samples were then separated by SDS-PAGE (4%–12% gel) (Bio-Rad) and analyzed by western blotting using antibodies recognizing Rab6a (Thermo Fisher, catalog #11420-1-AP, diluted 1:1,000), BICD2 (Abcam, ab117818, 1:1,000), beta-actin (Sigma, A5441, 1:5,000), FLAG (Sigma, F1804, 1:1,000; Sigma, A8592, 1:1,000; or Invitrogen, MA1-142, 1:1,000), or HA (Cell Signaling, 3724, 1:1,000; Cell Signaling, 2999, 1:1,000; or Roche, 54732500, 1:1,000). Secondary horseradish peroxidase-conjugated antisera recognizing rabbit, rat, or mouse antibodies as appropriate (Jackson ImmunoResearch, 711-035-152, 712-036-150, 115-035-146) were used at 1:5,000 dilution in 5% non-fat milk unless specified otherwise. The blots were developed with SuperSignal West Pico or Femto Chemiluminescent substrate (Pierce) and were visualized by using a film processor (Fujifilm).

### PLA

HeLa cells (0.35 × 10^5^ cells per well) were plated in 24-well plates containing glass coverslips 48 h prior to infection. Approximately 6 h later, cells were transfected with 6.7 nM of indicated siRNA (Table S1) as described above. At 40 h–48 h after transfection, cells were infected with PsVs at MOI of ~40. As indicated, dimethylsulfoxide (DMSO) (0.2% as final concentration), 100 nM BafA, or 2 µM XXI was added to the medium 30 min prior to infection and kept in the medium for the duration of the experiment. At indicated times post-infection, cells were fixed with 4% paraformaldehyde (Electron Microscopy Sciences) at room temperature (RT) for 12 min, permeabilized with 1% saponin (Sigma-Aldrich) at RT for 35 min–40 min, and blocked using DMEM10 at RT for 1 h–1.5 h. Cells were then incubated overnight at 4°C with a pair of mouse and rabbit antibodies: a mouse antibody recognizing L1 (BD Biosciences, 554171, 1:1,000 when used with anti-TGN46, 1:500 when used with anti-dynein, 1:200 when used with other antibodies), Rab6a (Sigma, WH0005870M1, 1:200), FLAG to detect FLAG-tagged L2 (Sigma, F1804, 1:1,000), and a rabbit antibody recognizing cellular proteins or epitope tags (anti-EEA1, Cell Signaling Technology, 2411, 1:75; anti-TGN46, Abcam, ab50595, 1:600; anti-GM130, Abcam, ab52649, 1:200; anti-Rab6a, Abcam, ab271094, 1:200; anti-BICD2, Abcam, ab117818, 1:200; anti-dynein, Invitrogen, PA5-89505, 1:200). PLA was performed with Duolink reagents (Sigma Aldrich) according to the manufacturer’s instructions as described (60). Briefly, cells were incubated in a humidified chamber at 37°C with a pair of PLA antibody probes (mouse and rabbit) for 75 min, with ligation mixture for 45 min, and then with amplification mixture for 3 h, followed by series of washes. Nuclei were stained with 4′,6-diamidino-2-phenylindole (DAPI). Cellular fluorescence was imaged using the Zeiss LSM980 confocal microscope. Images were processed using a Zeiss Zen software version 3.1 and quantified using Image J Fiji version 2.3.0/1.53f.

### Protein purification

HEK293TT cells were seeded in a 15 cm plate and transfected with plasmids expressing FLAG-BICD2 (30), HA-tagged CA Rab6a, or HA-tagged DN Rab6a. After 48 h, cells were washed three times with DPBS, harvested, and lysed in HEPES-NaCl (HN) buffer (50 mM HEPES and 150 mM NaCl) containing 1% Triton X-100 and protease inhibitor cocktail (Thermo Fisher Scientific). Cells were incubated on a rotator for 20 min at 4°C then centrifuged for 15 min at 17,000 × *g*. The resulting supernatant was incubated with anti-FLAG M2 or anti-HA agarose beads (MilliporeSigma, A2220; Pierce, 26181) for 2 h at 4°C. The beads were then washed once with the lysis buffer, twice with the lysis buffer containing 1 mM ATP to remove contaminating proteins, then once with HN buffer containing 0.1% Triton X-100. All buffers contain protease inhibitor cocktail. The proteins were then eluted twice with 3×FLAG or HA peptides in HN buffer containing 0.1% Triton X-100 at RT for 30 min. The quality of the purified proteins was analyzed by SDS-PAGE and SimplyBlue SafeStain (Invitrogen), and their quantity was determined using the BCA Protein Assay Kits (Pierce, 23225).

### Peptide pulldown

Peptides were purchased from GenScript or Promega and dissolved in DMSO. Peptide stocks were diluted to 5 mg/mL and stored at −80°C. Purified proteins (~20 nM of FLAG-BICD2 or HA-tagged CA or DN Rab6a, unless otherwise specified) were incubated with 5 µg of biotinylated peptide in HN buffer containing 0.1% Triton, 1 mM dithiothreitol (DTT), and protease inhibitor cocktail (Thermo Fisher Scientific) for 2 h at 4°C with mild rotation. Thirty microliters of Pierce streptavidin magnetic beads (Thermo Fisher Scientific) was added to the samples and incubated for an additional hour at 4°C. The beads were then washed three times with HN buffer and eluted by incubation at 95°C for 10 min in SDS sample buffer with 2-mercaptoethanol. Precipitated proteins were detected by western blot analysis.

### Co-immunoprecipitation of BICD2 and L2

HeLa cells were grown to ~70% confluency in 10 cm plates before siRNA transfection. Forty to 48 hpt, cells were infected with HPV16 PsV at the MOI of ~1. At 16 hpi, the cells were washed three times with DPBS. The second PBS wash contained 300 mM NaCl to remove extracellular HPV. Cells were lysed in 400 µL of modified RIPA buffer (50 mM Tris [pH 7.4], 150 mM NaCl, 0.25% sodium deoxycholate, 1% NP40, and 1 mM EDTA) containing 1 mM phenylmethylsulfonyl fluoride (PMSF). Cells were incubated on ice for 10 min followed by centrifugation at 16,100 × *g* for 10 min. After centrifugation, 10% of the supernatants were taken for input, and the remaining supernatants were incubated with antibody recognizing BICD2 or control IgG overnight at 4°C. Pierce protein A/G agarose beads (Thermo Fisher Scientific) were then added to the samples for 30 min at 4°C, followed by three washes with the RIPA buffer and eluted by incubation at 95°C for 10 min in 5× SDS sample buffer with 2-mercaptoethanol. Samples were then analyzed by SDS-PAGE, and western blot analysis carried out as described above.

### Determining HPV PsV infectivity in cells transiently expressing CA or DN Rab6a

293TT cells (0.35 × 10^5^ cells per plate) were plated in 24-well plates 16 h–20 h prior to transfection of plasmids encoding HA-tagged CA or DN Rab6a, or the empty vector as control (Takara, 632105). Forty-eight hours after transfection, cells were infected with one-half to one-third of the amount of PsV used for infectivity studies in HeLa cells (resulting in the infection of ~2/3 of the cells). Forty-eight hours post-infection, cells were fixed with 4% paraformaldehyde, permeabilized with 1% saponin, and blocked with 3% bovine serum albumin (BSA). Cells were then stained using PE-conjugated antibodies recognizing HA (MACS Molecular, 130-120-717, 1:1,000), followed by three to four times of washes using DPBS containing 0.1% Tween-20. HA-tagged Rab protein abundance and HPV PsV infectivity were determined by flow cytometry to measure fluorescence intensity produced by PE-stained proteins and GFP.

### Immunofluorescence

HeLa cells were seeded onto glass coverslips in a six-well plate and treated as indicated. Cells were washed three times with PBS, fixed in 4% paraformaldehyde for 20 min at RT then washed four times with PBS. Permeabilization was carried out for 20 min at RT with Tris-buffered saline (TBS)/0.2% Triton X-100/3% BSA (TBS-TB) followed by three washes with TBS-T (TBS with 0.1% Tween-20). The cells were blocked with TBS-TB for 1 h at RT. Primary antibodies (anti-BICD2, Novus Bio, NBP 2-43683, 1:200; anti-TGN46, Proteintech, 13573-1-AP, 1:2,000) were diluted in TBS-TB and incubated with the coverslips overnight at 4°C. Secondary antibodies (goat anti-rabbit Alexa Fluor 488, Thermo Fisher Scientific, A11008, 1:2,000; goat anti-mouse Alexa Fluor 594, Thermo Fisher Scientific, A11032, 1:2,000) were diluted with TBS-TB and incubated with coverslips for 1 h at RT. Coverslips were mounted with mounting medium containing DAPI (Abcam, ab104139). Images were taken with confocal microscopy (Zeiss LSM 800 confocal laser scanning microscope with a Plan-Apochromat 40×/1.4 oil differential interference contrast M27 objective). Representative images were chosen out of three independent experiments.

### Construction of plasmids

Plasmids expressing HA-tagged Rab6a CA or DN variants were constructed as follows: CA and DN mutant Rab6a genes were amplified from enhanced green fluorescent protein (EGFP)-Rab6AQ72L (Addgene #49483) and EGFP-Rab6AT27N (Addgene, Plasmid #49484), respectively, using primers HA-Rab6a-BamHI-F (TGA ACC GTC AGA TCG CCT GGA GAA GGA TCC ATG TAC CCA TAC GAC GTT CCA GAT TAC GCT TCC ACG GGC GGA GAC TTC GGG AAT CCG) and Rab6a-EcoRI-R (GAA AAG CGC CTC CCC TAC CCG GTA GAA TTC TTA GCA GGA ACA GCC TCC TTC ACT GAC TGG TTG), and then introduced between BamHI and EcoRI sites of pRetroX-Tight-Pur vector (Takara, 632105). Resulting plasmids containing the desired mutation were confirmed by DNA sequencing.

For protein purification, primers HAR6a-pCMV-XhoI-F (TAC AAG CTA CTT GTT CTT TTT GCA CTC GAG GCC ACC ATG ATG TAC CCA TAC GAT GTT CCA GAT TAC GCT TCC ACG GGC GGA G) and HAR6a-pCMV-AgeI-R (GTA TCT TAT CAT GTC TGC TCG AAG CGG ACC GGT TTA GCA GGA ACA GCC TCC TTC ACT GAC TGG TTG) were used to amplify CA or DN HA-tagged Rab6a, then introduced between XhoI and AgeI sites of pCMV-FLAG vector (Takara, 632105). Resulting plasmids containing the desired mutation were confirmed by DNA sequencing.

### Cell cycle analysis

HeLa cells were transfected with control and Rab6a siRNA. At 40 hpt, cells were washed once with DPBS, and DMEM10 containing 10 µg/mL of Hoechst 33342 was added to the cells. After 30-min incubation at 37°C, cells were analyzed using flow cytometry.

### Statistical analyses

For comparisons of two groups, unpaired *t*-tests were applied. For comparisons of more than three groups, one-way analysis of variance (ANOVA) with the ordinary ANOVA test was performed. These analyses provide *P*-values for each comparison.

## SUPPLEMENTAL MATERIAL

Supplemental material mbio.02811-24-s0001.pdf Supplemental figures and table.

ASM does not own the copyrights to Supplemental Material that may be linked to, or accessed through, an article. The authors have granted ASM a non-exclusive, world-wide license to publish the Supplemental Material files. Please contact the corresponding author directly for reuse.
