# Supplementary material for: Rab6a enables BICD2/dynein-mediated trafficking of human papillomavirus from the trans-Golgi network during virus entry
Source: mBio. 2024 Oct 21;15(11):e02811-24. doi: 10.1128/mbio.02811-24 (PMC11559006; doi:10.1128/mbio.02811-24)
Supplement: Supplemental material — Supplemental figures and table. [file mbio.02811-24-s0001.pdf]

***Supplemental Material* for**

Rab6a enables BICD2/dynein-mediated trafficking of human papillomavirus from the *trans*-Golgi network during virus entry

Jeongjoon Choi[1], Kaitlyn Speckhart[2,3], Billy Tsai[2,3], and Daniel DiMaio[1,4,5,6,*]

[1]*Department of Genetics, Yale School of Medicine, PO Box 208005, New Haven, CT 06520-8005, USA*

[2]*Department of Cell and Developmental Biology, University of Michigan Medical School, 109 Zina Pitcher Place, Ann Arbor, MI 48109*

[3]*Cellular and Molecular Biology Program, University of Michigan Medical School*

[4]*Department of Therapeutic Radiology, Yale School of Medicine, PO Box 208040, New Haven, CT 06520-8040, USA*

[5]*Department of Molecular Biophysics & Biochemistry, Yale University, PO Box 208024, New Haven, CT 06520-8024, USA*

[6]*Yale Cancer Center, PO Box 208028, New Haven, CT 06520-8028, USA*

**Correspondence: daniel.dimaio@yale.edu*

Supporting Information contains followings:

Fig. S1 to S3 and figure legends

Table S1

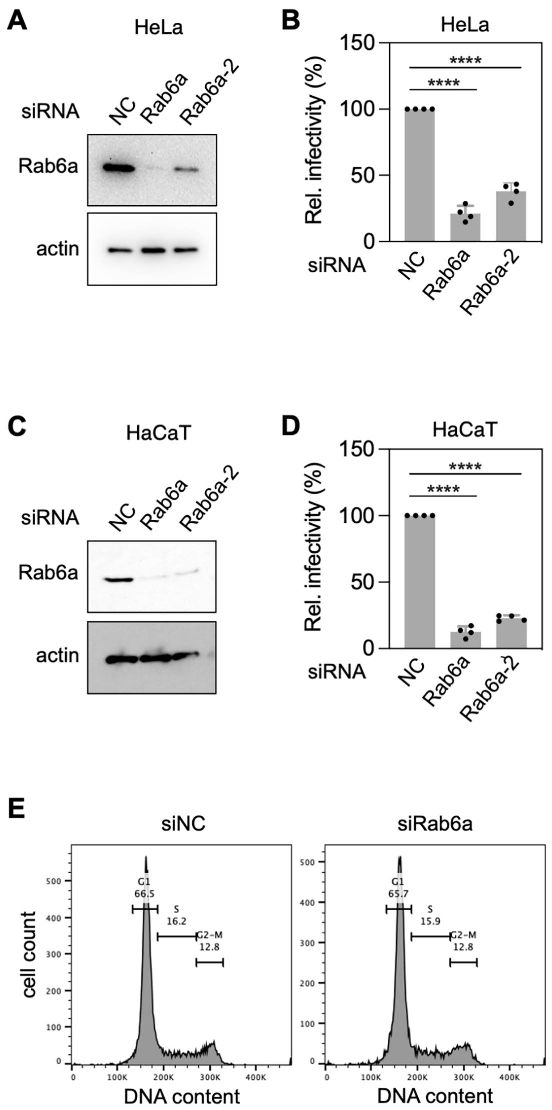

Fig. S1. Rab6a is required for HPV entry in both HeLa and HaCaT cells. (**A**) HeLa cells were transfected with siNC or two different siRNAs targeting Rab6a (siRab6a and siRab6a-2). At 48 h post transfection, cell extracts were prepared and subjected to western blot analysis using an antibody recognizing Rab9a or actin. (**B**) siRNA-treated cells as described in (**A**) were mock-infected or infected at the MOI of ~1 with HPV16 PsV L2-3XFLAG containing the GFP reporter plasmid. At 48 hpi, GFP fluorescence was determined by flow cytometry. The results are shown as percent relative infectivity (based on mean fluorescence intensity) normalized to siNC treated cells. Each dot shows the result of an individual experiment. Bars and error bars show mean and standard deviation, respectively. ****, $P < 0.0001$. (**C**) As in (**A**) except using HaCaT cells. (**D**) As in (**B**) except using HaCaT cells. (**E**) HeLa S3 cells were transfected with siNC or siRNAs targeting Rab6a. At 48 h post transfection, cellular DNA was stained with Hoechst 33342 and Hoechst fluorescence was determined by flow cytometry. Similar results were obtained in two independent experiments. Representative histograms are shown.

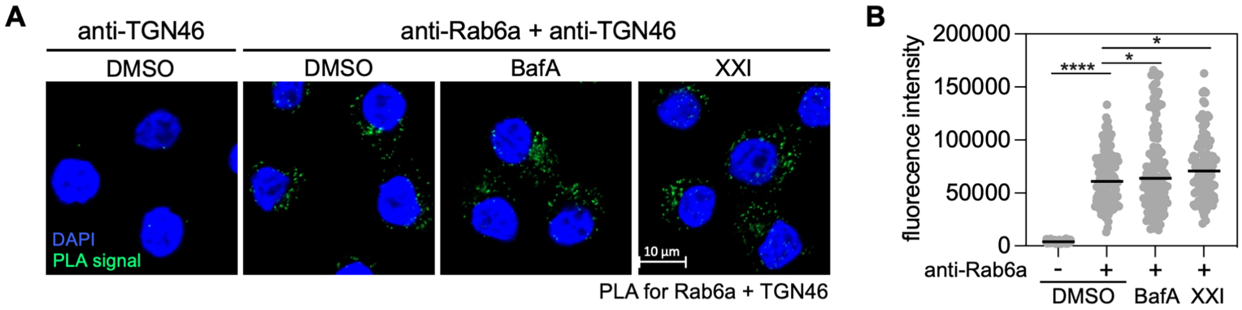

Fig. S2. BafilomycinA1 and XXI do not cause redistribution of Rab6a from the TGN. (**A**) HeLa cells were treated with DMSO, BafA1, or γ-secretase inhibitor XXI. At 16.5 h after drug treatment, PLA was performed with antibodies recognizing Rab6a and TGN46. PLA signals are green; nuclei are blue. Similar results were obtained in two independent experiments. (**B**) The fluorescence of PLA signals was determined from multiple images obtained as in (**A**). Each dot represents an individual cell (n>40) and black horizontal lines indicate the mean value of the analyzed population in each group. *, $P < 0.05$; ****, $P < 0.0001$. The graph shows results of a representative experiment.

```
HPV5     490 VVIIHPHDSTGDFYLHPSLH-RRKRKRKYL
                       .:******** :  ***:::
HPV16    434 SPQYTIIADAGDFYLHPSYYMLRKRRKRLPYFFSDVSLAA
             * ** *  .. .:** * **:: *:***:****:*  :**
HPV18    424 STQY-IGIHGTHYYLWPLYYFIPKKRKRVPYFFADGFVAA
```

Fig. S3. The C-terminal segments of L2 from different types of HPV bind Rab6a. C-terminal L2 segments of HPV5 and HPV18 were aligned with HPV16 L2 (positions 434-473). The asterisks indicate identity, colons indicate conservative substitutions, and periods indicate semi-conservative substitutions.

Table S1. siRNAs used in this study.[a]

siRNA	identifier
siGENOME Human RAB6A siRNA<br>(shown as siRab6a in this study)	D-008975-04-0005
ON-TARGETplus Human RAB6A siRNA<br>(shown as siRab6a-2 in this study)	J-008975-08-0002
ON-TARGETplus Human RAB33B siRNA<br>(SMARTPool)<br>(shown as siRab33b in this study)	L-008909-00-0005
ON-TARGETplus Human RGP1 siRNA (SMARTPool)<br>(shown as siRgp1 in this study)	L-021128-02-0005
ON-TARGETplus Human RIC1 siRNA (SMARTPool)<br>(shown as siRic1 in this study)	L-026110-02-0005
ON-TARGETplus Human Rab7A and Rab7B siRNA<br>(SMARTPool)<br>(shown as siRab7 in this study)	L-010388-00-0005<br>L-018225-00-0005
ON-TARGETplus Non-targeting Control Pool<br>(shown as siNC in this study)	D-001810-10-20

[a] All siRNAs were purchased from Dharmacon, Inc.
